# Supplementary material for: Electrodeposited magnetic nanoporous membrane for high-yield and high-throughput immunocapture of extracellular vesicles and lipoproteins
Source: Commun Biol. 2022 Dec 10;5:1358. doi: 10.1038/s42003-022-04321-9 (PMC9741596; doi:10.1038/s42003-022-04321-9)
Supplement: Supplementary file 2 — Supporting Information [file 42003_2022_4321_MOESM2_ESM.pdf]

Supporting Information

Electrodeposited Magnetic Nanoporous Membrane (MNM) with a Multi-Edge Superparamagnetic Heterogeneous Wedge Junction for High-Yield and High-Throughput Immunocapture of Specific Extracellular Vesicles and Lipoproteins

Chenguang Zhang[1,#], Xiaoye Huo[1,#], Yini Zhu[2], James N. Higginbotham[3], Zheng Cao[3], Xin Lu[2], Jeffrey L. Franklin[3,4], Kasey C. Vickers[3], Robert J. Coffey[3,4], Satyajyoti Senapati[1], Ceming Wang[5,*], Hsueh-Chia Chang[1,*]

[1] Department of Chemical and Biomolecular Engineering, University of Notre Dame, Notre Dame, IN 46556, USA.

[2] Department of Biology, University of Notre Dame, Notre Dame, IN 46556, USA.

[3] Department of Medicine, Vanderbilt University Medical Center, Nashville, TN 37232, USA.

[4] Department of Cell and Developmental Biology, Vanderbilt University School of Medicine, Nashville, TN 37232, USA.

[5] Aopia Biosciences, 31351 Medallion Dr, Hayward, CA 94544, USA.

[#] These authors contributed equally: Chenguang Zhang, Xiaoye Huo.

corresponding author email: cwang9@nd.edu, hchang@nd.edu

Supplementary Note 1. Simulation of Heterogeneous Superparamagnetic Nano-Junction

In this work, COMSOL has been used to model and simulate different nanopore structures to estimate the magnetic flux density and its gradient. The magnetic force on the nanomagnetic beads is proportional to the norm square of the magnetic flux density gradient. A two-dimensional (2D) axial-symmetry geometry model has been made with the Magnetic Fields, No Currents interface in the AC/DC module. The schematic of the model is as shown in Figure 2. The modeled nanopores consist of 200nm NiFe and 80nm Au. Different ideal geometries are defined for both sputtered and electroplated pores. In this model, the axial symmetry boundary is along r = 0, and the axial boundaries condition at r=10μm are set to be magnetically insulated. The software built-in NiFe B-H curve is used. All the other material

properties are chosen to be linear and isotropic. In addition, a static magnetic flux density of 0.5T is applied at the far boundary of the model. The simulation is conducted with a physics-controlled meshing of extremely fine elements. The 2D axial symmetry analysis uses a low processor memory and faster solution convergence.

Supplementary Note 2. Additional Characterization of Electroplated/Sputtered Magnetic Nanoporous Membrane

We examined the structure of the NiFe layer by both SEM (Figure S1a, b) and XRD (Figure S1c, d). For XRD, a peak around 2θ=44.5° was investigated to determine the grain size of both electroplated and sputtered NiFe. No significant difference in the minimum grain size is found between the two (~30nm). However, large grain boundaries can be observed on the sputtered NiFe but not the electroplated one under SEM. Such grain boundaries will result in lower magnetic permeability of the NiFe, which leads to lower magnetic force on the nanobeads.

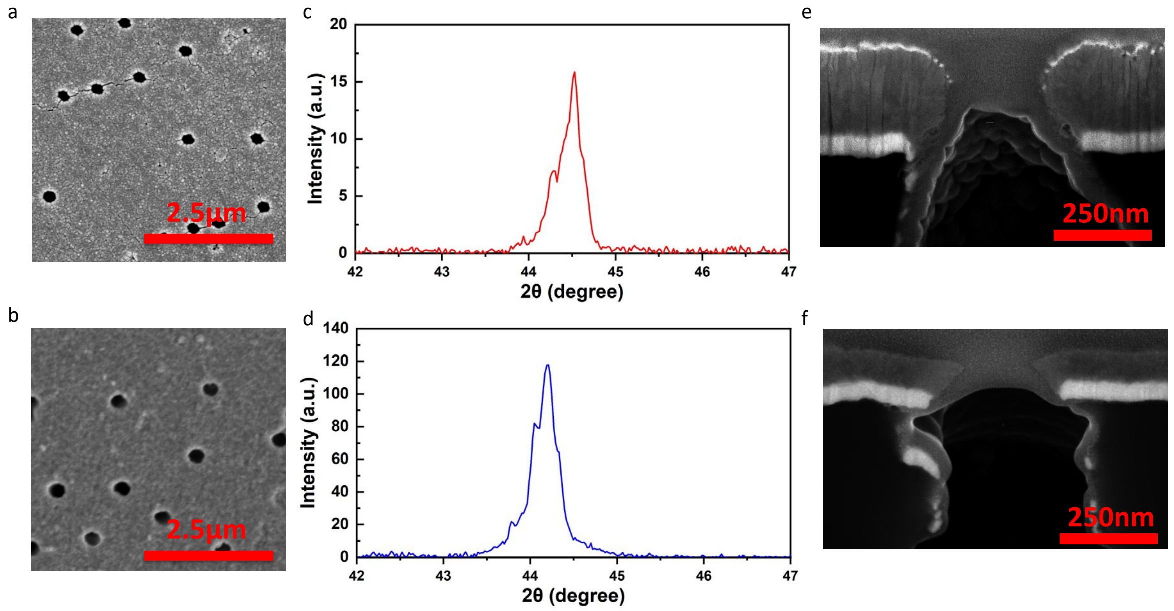

Figure S1. Surface characterization of the electroplated and sputtered membrane. SEM images of the a) sputtered membrane and the b) electroplated membrane. HiRes-XRD of the c) sputtered membrane and the d) electroplated membrane. Cross section SEM images of the e) sputtered membrane and the f) electroplated membrane.

Supplementary Note 3. Instrumentation and Workflow of the Magnetic Nanoporous Membrane Immunocapturing

The experimental setup for testing the immunocapturing of magnetic nanoporous membrane is shown in Figure S2a. Briefly, the MNM was fixed between two SLA-3D-printed membrane holders (Figure S2c). We designed 1mm openings on the holders as a liquid inlet and outlet. Notches on the outside were milled to fit in magnets and minimize the distance from the magnets to the membrane. The inside chamber has a diameter of 2cm and a height of 100μm for minimal liquid retention. Figure S2b shows the assembled MNM device. A syringe pump is programmed to inject solution at a constant rate. The flow-through is collected from the outlet. Experiments requiring downstream biological analysis were performed inside the biological safety cabinet.

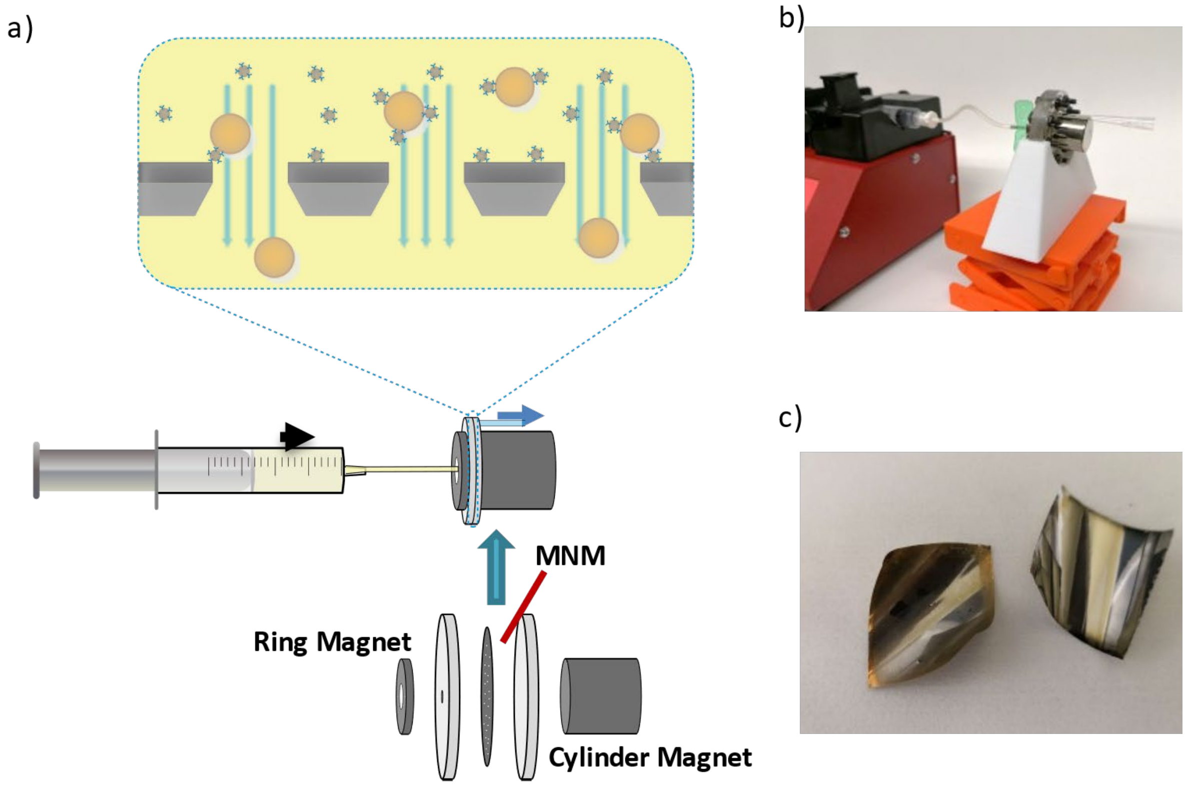

Figure S2. Schematic and picture of the MNM device. a) Schematic of the bead capture device.The sample was injected into the chamber of the MNM device with syringe and pump. The magnetic membrane was sandwiched between two 3D-printed chips. The device was assembled between two magnets. The magnetic beads were captured onto the edge of the

nanopores as highlighted. b) Picture of the actual experimental setup. (c) Picture of the electroplated nanoporous membranes.

Supplementary Note 4. Proof-of-Concept for MNM EV Immunocapturing

A proof-of-concept extracellular vesicle immunocapture experiment with MNM was also carried out with anti-CD9, CD64, CD81 functionalized nanobeads. 100µL mouse CD9, CD64, CD81 nanobeads were mixed with 100µL plasma from healthy mice models and incubated at room temperature for 1 hour. The solution was then diluted to 1mL with 1×PBS and passed through 450nm electroplated magnetic nanoporous membrane at 1mL/hr, followed by flushing with 1mL 1×PBS to remove beads adsorbed elsewhere in the apparatus instead of the MNM, and also to remove the residual sample solution in the chamber. SEM image (Figure 3d) shows mouse plasma exosome docked with nanobeads captured near the nanopore. We benchmarked the capture yield by lysing the exosomes of the MNM trapped nanobeads and those by micro dynabeads (Figure S3) and conducting qRT-PCR of microRNA miR-21.

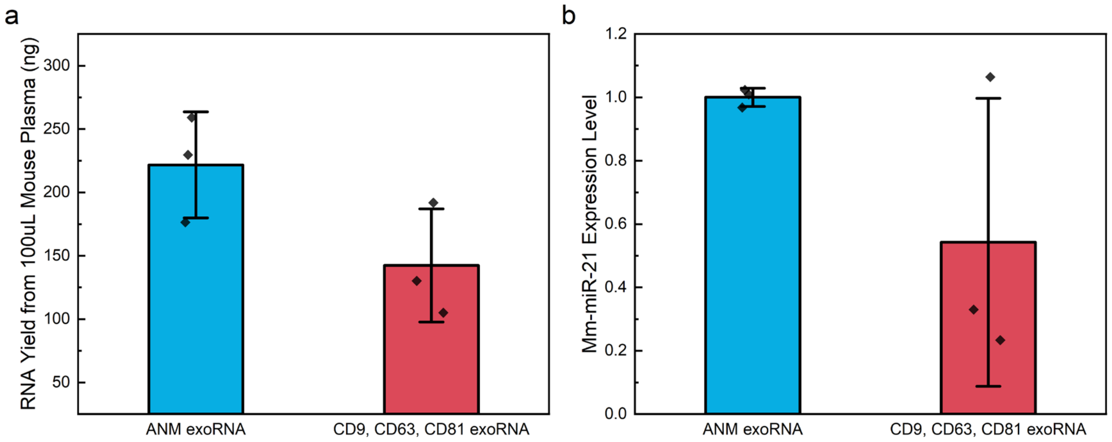

Figure S3. RNA quantification of the immunocaptured EV with MNM. a) Yield of RNA after the fractionation of EV with asymmetric nanoporous membrane and then after the CD9, CD63 and CD81 immunocapture with MNM. b) qRT-PCR result of miR-21 from the two extracted samples. Error bars (n=3) indicate the standard deviation (SD) in each plot.

Supplementary Note 5. HDL immunocapture using commercial kits

For nanobeads, the µColumns (Milyteni Biotech) were used for the bead capture. Anti-ApoA1 antibodies (Abcam, ab52945, rabbit monoclonal to ApoA1) were mixed with HDL samples and incubated for 30min, then anti-rabbit IgG microBeads (Milyteni Biotech) were added and incubated for 1hr. After the HDLs were immunocaptured by the magnetic beads, the magnetic separation was performed as follows. The µColumns (Milyteni Biotech) were placed on the µMACS™ separator or our custom ring magnet rack and were rinsed with 500µL rinsing solution (PBS, 0.5% BSA, and 2 mM EDTA). Then the sample was applied onto the column. Then 3×500µL rinsing buffer was used to wash the column. The flow-through was collected under the column. Then the column was removed from the stand, and 300µL buffer was used to flush the microbeads out by firmly pushing the plunger into the column. The results are shown in Figure S4a.

We also tested Dynabeads™ as the most popular microbead protocol. Anti-ApoA1 antibodies were mixed with HDL samples and incubated for 30min, then 50µL microbeads(Thermofisher, Dynabeads™, Protein G) were washed by being placed on a magnetic separation stand (Promega, MagneSphere®), then the Ab-Ag complex was added, followed by incubation. The Dynabeads-Ab-Ag complex was washed 3 times, using 200µL washing buffer for each wash. To elute the antigen, the Dynabeads-Ab-Ag complex was pulled down by the magnet and suspended in 20µl elution buffer. As shown in Figure S4b, the capture rate saturated after 16 hours at 50%.

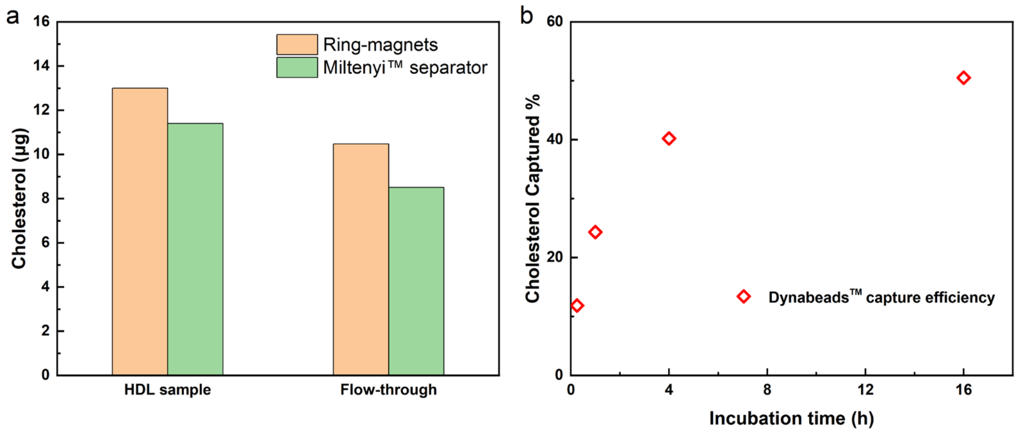

Figure S4. HDL capture rate of other technologies. a) HDL(Cholesterol) capture rate of the µColumn (Milyteni Biotech, MACS™). The cholesterol was quantified before and after the MNM capture in the flowthrough. With the same column, both ring-magnets and Milyteni separator were used to magnetize the column. The ring-magnet which we put the column into was a economical substitution to the MACS™ magnetic separator. b) HDL(Cholesterol) capture rate of Thermofisher Dynabeads™ with different incubation time. The protocol of the commercial kit indicates an incubation time of 25min which results in capture rate as low as 11.9%, and an increased incubation time of as long as 16h obtained only 50.5% yield.

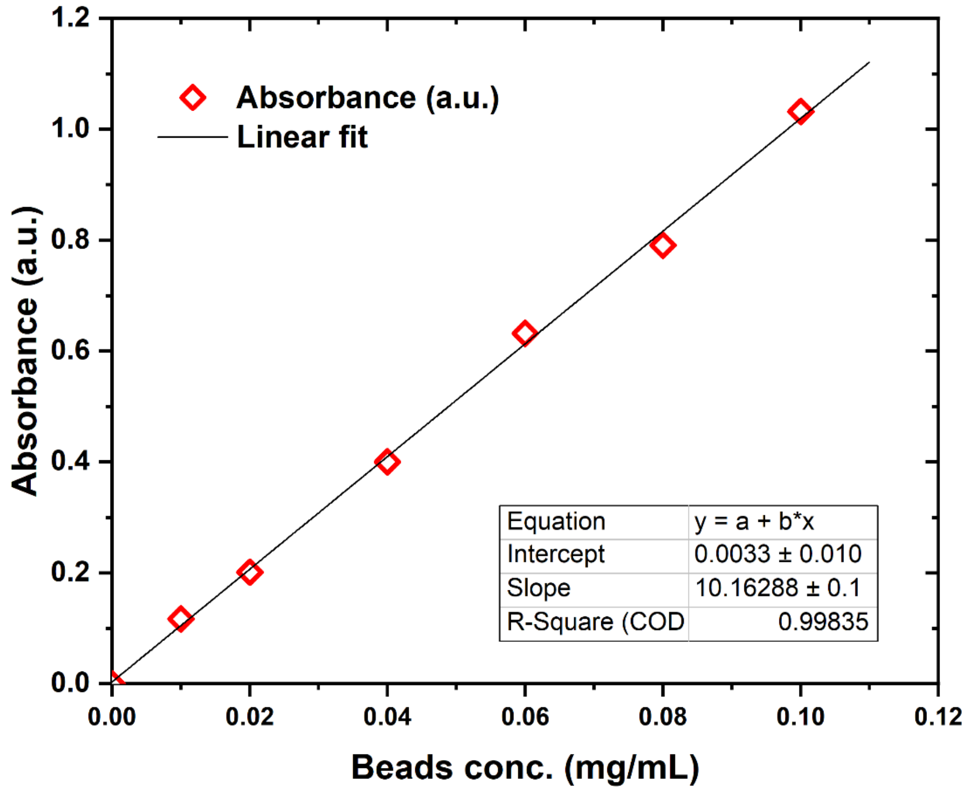

Figure S5. Calibration curve of the beads concentration. The absorbance was measured at the wavelength of 288nm, and it increases linearly with the beads concentration.

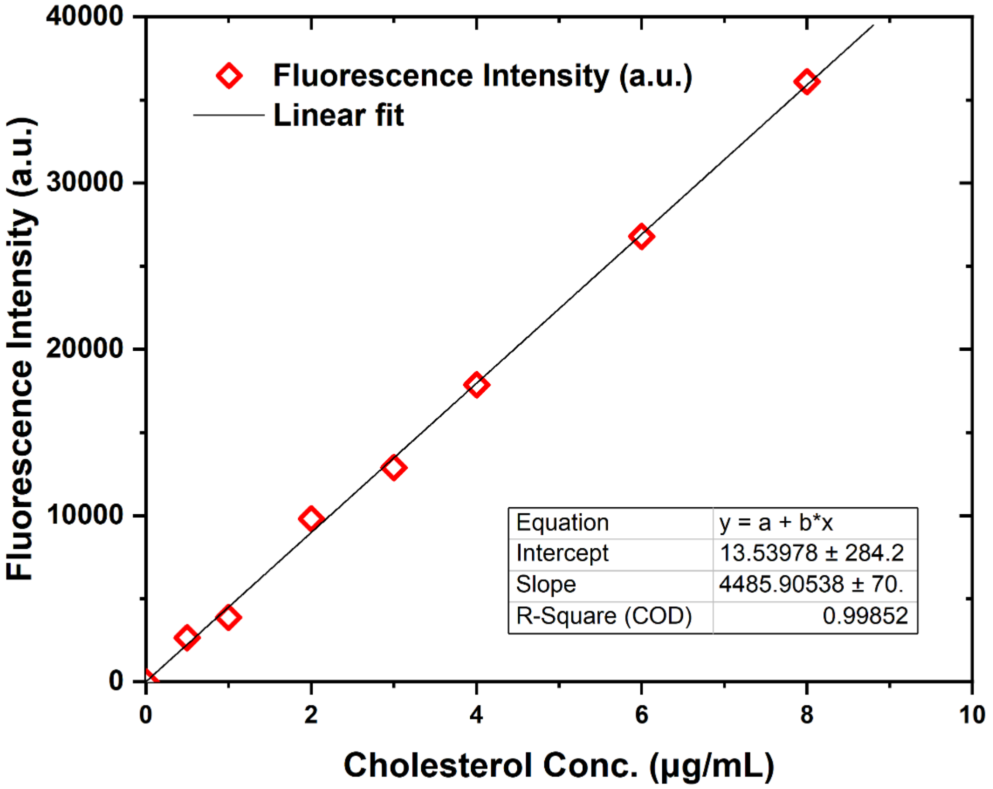

Figure S6. Calibration curve of the cholesterol assay. The fluorescence intensity was measured in a fluorescence microplate reader using excitation at 545 nm and emission detection at 590 nm, and it increases linearly with the cholesterol concentration.

Supplementary Note 6. Electroplating Solution

The compounds used for electroplating electrolyte are described in Table S1. The compound was mixed with DI water and solved at 40℃ with agitation for 1 hour. Sodium hydroxide was used to adjust the pH of the solution to 3.0.

**Compound**	**Amount**[g/ 100ml]
$NiSO_4.6H_2O$	28.9
$FeSO_4.7H_2O$	6.4
H_3BO_3	4
5-Sulfosalicylic acid dihydrate	0.890
1,3,(6,7)-Naphthalenetrisulfonic acid trisodium salt hydrate	0.304

Table S1. Recipe for the electroplating solution. The pH-value was adjusted to 3.0 using sodium hydroxide.

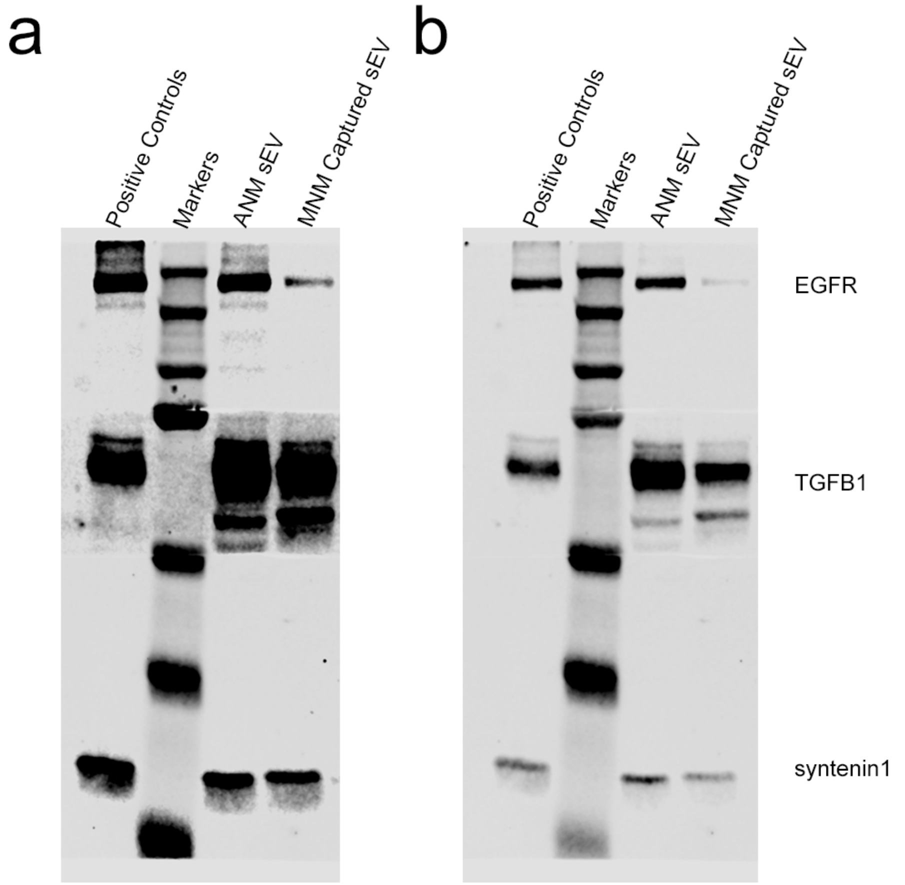

Figure S7. Uncropped and unedited gel images of the isolated EVs from the DiFi cell line. The Odyssey exposure was set to (a) overexposed and (b) 10 min respectively. The gel was cut into 3 pieces the top was probed for EGFR, the middle panel was probed for TGFBI and the bottom was probed for syntenin1.
